# Fracture Behavior of AA7075-AA6061 and AA7075-Cu Friction-Stir Welded Joints Containing Blunt V-Notches under Opening-Mode Loading

**DOI:** 10.3390/ma16051757

**Published:** 2023-02-21

**Authors:** Ali Reza Torabi, Moslem Mirzavand, Behnam Saboori, Sergio Cicero

**Affiliations:** 1 Fracture Research Laboratory, Faculty of New Sciences and Technologies, University of Tehran, Tehran 14395-1561, Iran; 2 Center of Excellence in Experimental Solid Mechanics and Dynamics, Fatigue and Fracture Research Laboratory, School of Mechanical Engineering, Iran University of Science and Technology, Tehran 16846, Iran; 3 Laboratorio de la División de Ciencia e Ingeniería de los Materiales (LADICIM), Departamento de Ciencia e Ingeniería del Terreno y de los Materiales, Universidad de Cantabria, 39005 Santander, Spain

**Keywords:** ductile fracture, V-notch, friction-stir welding, equivalent material concept, mean stress criterion, maximum tangential stress criterion

## Abstract

The purpose of this study is to predict the load-bearing capacity (LBC) of fracture specimens containing V-notched friction-stir welded (FSWed) joints of AA7075-Cu and AA7075-AA6061 materials and subjected to mode I loading conditions. Due to the resulting elastic-plastic behavior and the corresponding development of significant plastic deformations, the fracture analysis of the FSWed alloys requires elastic-plastic fracture criteria, which are complex and time-consuming. Thus, in this study, the equivalent material concept (EMC) is applied, equating the actual AA7075-AA6061 and AA7075-Cu materials to equivalent virtual brittle materials. Then, two brittle fracture criteria, the maximum tangential stress (MTS) and mean stress (MS), are utilized to estimate the LBC of the V-notched FSWed parts. The comparison between the experimental results and the theoretical predictions reveals that both fracture criteria, in combination with EMC, can accurately predict the LBC in the analyzed components.

## 1. Introduction

Unlike the case of cracks, designers often include notches as practical structural solutions in engineering structures. The introduction of a notch in a given structural part concentrates stresses around the notch tip, leading to a reduction of the overall strength of this notched part against externally applied loads. In order to ensure the strength and structural integrity of notched components, it is necessary to analyze their fracture behavior in accordance with the type of loading being applied (e.g., opening loads, shear loads, mixed modes, etc.).

Aluminum and copper alloys are widely used in industry. In the past, researchers have studied the mechanical properties of these alloys, such as tensile strength and bending strength. However, the fracture (and fatigue) behavior of friction-stir welded (FSWed) joints made of such alloys has not been investigated so profusely, especially when the effect of notches is included in the research. In the following, some research conducted in these fields is reviewed.

Sutton et al. [1] investigated the mode I fracture behavior at the joint of aluminum alloys butt-bonded by friction-stir welding (FSW). In that study, they used the critical crack opening displacement at a given distance from the crack tip to explore the fracture behavior of the joint, and showed that this crack opening displacement measurement is able to provide the quantitative value of the corresponding fracture toughness. Moreira et al. [2] characterized the mechanical behavior of friction stir welding joints of AA6061-T6 with AA6082-T6, showing that the FSWed joint develops intermediate mechanical properties when compared with the two base materials. Moreover, Zadpour et al. [3] studied the properties of welded joints of AA7075-T6 and AA2029-T3 alloys. Reynolds [4] assessed the fracture toughness of the aluminum-lithium alloy 2195-T8 friction stir weld using compact-tension samples. Mokhtar et al. [5] investigated the mode I fracture toughness and the fatigue crack growth behavior of an FSWed AA6061 sheet. Sutton et al. [6] evaluated the mode I fracture behavior and the microstructure in AA2024-T3 friction stir welds. Aliha et al. [7] experimentally investigated the fracture toughness of AA5083-Cu FSWed joints containing notches and subjected them to pure mode I loading. Yan et al. [8] studied the crack growth behavior in AA2024 and AA2524 friction stir welds. Syafiq et al. [9] measured the fracture toughness of AA6061 and AA5053 FSWed joints. Moreover, Fratini et al. [10] explored the fatigue crack growth behavior in FSWed joints of AA2024-T351.

Concerning the effect of welding parameters on the mechanical performance of the welded joints, Pouget and Reynolds [11] explored the effect of residual stresses and microstructure on the fatigue behavior of AA2050 FSWed joints. Bahemmat et al. [12] investigated the effect of welding parameters such as the pin profile and the rotation speed on microhardness, microstructure, elongation, and tensile strength of AA7075-T6 FSWed joints. Furthermore, Aliha et al. [13] optimized the process parameters of an aluminum-aluminum dissimilar joint (i.e., 5XXX and 6XXX series) welded by FSW. Hatamleh et al. [14] studied the fatigue crack growth behavior in AA7075 under different loading conditions. Also, Moreira et al. [15] evaluated and compared the fatigue crack growth behavior of friction stir welds of AA6082-T6 and AA6061-T6 subjected to mode I loading conditions. They used compact-tension specimens containing an edge crack at different locations (e.g., at weld material and parallel to the weld line, at heat-affected zone and parallel to the weld line, etc.). Reynolds et al. [16] also investigated in FSWed AA7050 the relations between different welding parameters and the resulting temperature history and hardness distribution. Alavi Nia et al. [17] studied the effect of welding parameters on the fracture toughness and the fatigue behavior of FSWed copper sheets. Moghadam and Farhangdoost [18] studied the effect of pin advancing and rotating speeds on the fracture behavior of AA2024 friction stir welds. The results of their research showed that the tool speed affects both the fatigue crack growth rate and fracture toughness. The damage mechanics-based approach and machine learning methods have also been used for fatigue analysis [19,20].

Regarding the assessment of notched components under non-linear elastic conditions, Torabi [21] investigated the load-bearing capacity (LBC) of ductile steel samples weakened by V-shaped notches using the equivalent material concept (EMC). The theoretical estimations derived in that research were in very good agreement with the experimental results. Also, the success of EMC in predicting the LBC values was confirmed for two regimes of plasticity (large and moderate) around the fracture area. Torabi et al. [22] used the EMC in combination with two fracture criteria (mean stress- MS and point stress- PS) in order to predict the initiation of cracking at the tip of U-shaped notches made of AA7075-T6 and AA6061-T6. The results showed that the two mixed criteria (EMC-MS and EMC-PS) could successfully predict the experimental results.

When dealing with mixed-mode loading conditions, Sutton et al. [23] studied the microstructure and fracture of AA2524 welded by the FSW method under mixed-mode I/II loading. Also, Torabi et al. [24] investigated the fracture of AA7075 and AA6061 joints welded by FSW in a cracked semicircular bending specimen under mixed mode I/II loading. Using the EMC in combination with the maximum tangential stress (MTS) criterion and the mean stress (MS) criterion, Torabi et al. [25] assessed the LBC of FSWed joints of AA7075-AA7075 weakened by notches under mixed mode I/II loading. In their research, the small difference between the theoretical estimations and the experimental results showed that the EMC in combination with brittle fracture criteria could be used to estimate the LBC of notched specimens subjected to mixed mode I/II loading. Furthermore, Torabi et al. [26] studied the fracture of FSWed joints of AA2024 and AA7075 containing notches under mixed mode I/II loading. They demonstrated that the difference between the MTS criterion and the MS criterion is small, and also that the EMC in combination with the brittle fracture criteria could be used to predict the LBC of the joints.

Following the previous research works mentioned above, it is intended in the present study to evaluate the LBC of AA7075-AA6061 and AA7075-Cu FSWed joints containing round-tip V-notches subjected to mode I loading. This study involves fracture experiments on notched semi-circular bend (SCB) specimens and theoretical fracture predictions derived from the application of both the MTS and the MS criteria in combination with the EMC.

## 2. Materials and Methods

### 2.1. Experimental Program

**Figure 1:**
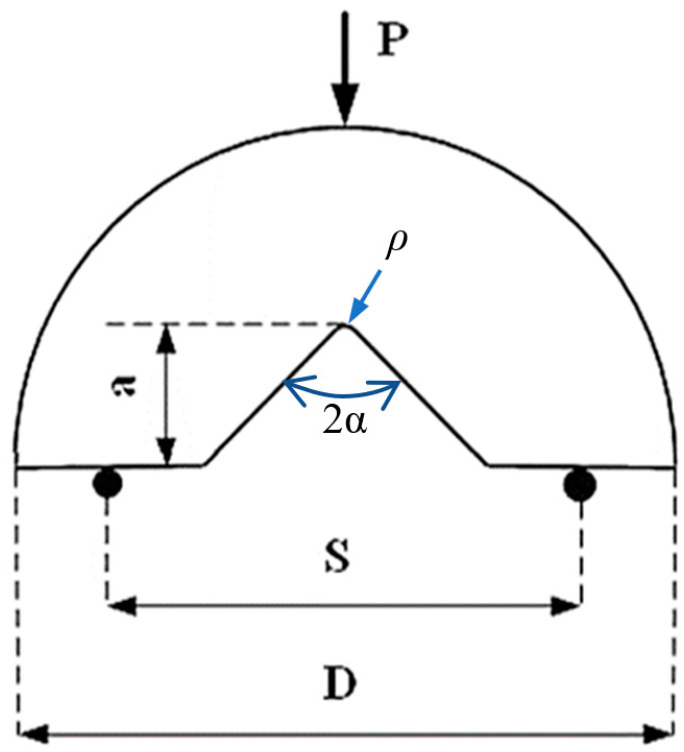
Geometry and dimensions of the V-notched semi-circular specimens under three-point bending.

In this research, fracture tests under mode I loading were performed on semi-circular bend (SCB) specimens containing V-shaped notches, according to the geometry of Figure 1. *P* is the applied load, *a* is the notch length, *2α* is the opening angle of the notch, *ρ* is the notch tip radius, *S* is the span and *D* is the diameter.

**Figure 2:**
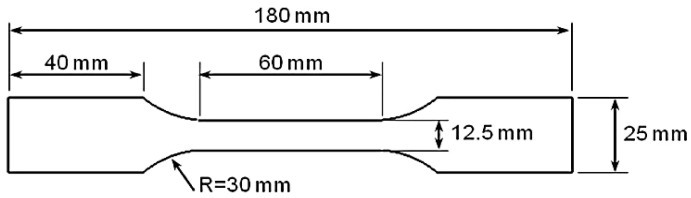
Geometry of the tensile specimens (*t* = 3 mm).

After cutting the AA7075, AA6061 and Cu primary sheets, they were subsequently welded to each other (AA7075-Cu, AA7075-AA6061) through friction stir welding (FSW). For welding, the rotational and linear speeds of welding were set in the computer numerical control milling machine. The pin was then placed on the seam between the two corresponding pieces applying a linear speed of 20 mm/min and a rotational speed of 600 rpm, which creates friction leading to the subsequent connection between the two pieces being joined. The width of the weld bead, which strongly affects the fracture behavior, is 20 mm. Post-weld solution-aging heat treatment was applied in order to improve the mechanical properties of the FSWed joints. To extract the geometry of the specimens from the welded sheets, semi-circular geometries with V-shaped notches of depth *a* = 15 mm were created with a cutting machine. Twelve semi-circular combinations of materials and geometries were finally manufactured, combining different notch angles and notch tip radii. Six of the combinations correspond to AA7075-AA6061 specimens, and six of them to AA7075-Cu specimens. Additionally, for each combination, 3 nominally identical specimens were prepared, leading to a total amount of 36 specimens. More precisely, four notch angles were considered (*2α* = 0, 30, 45, 60 degrees), whereas the notch tip radii took values of *ρ* = 1, 2, 4 mm. Here, it is important to note that *2α* = 0 notches are often referred to as U-notches. The diameter, the span and the thickness were fixed at *D* = 60 mm, *2S* = 40 mm, and *t* = 3 mm, respectively. In addition, dumbbell-shaped tensile samples for AA7075-T6, AA6061-T6, Cu, and both AA7075-Cu and AA7075-AA6061 weld bead materials, with three repetitions each, were prepared. The tensile tests were performed according to ASTM-E8 standard [27], with the geometry of the specimens being shown in Figure 2.

**Figure 3:**
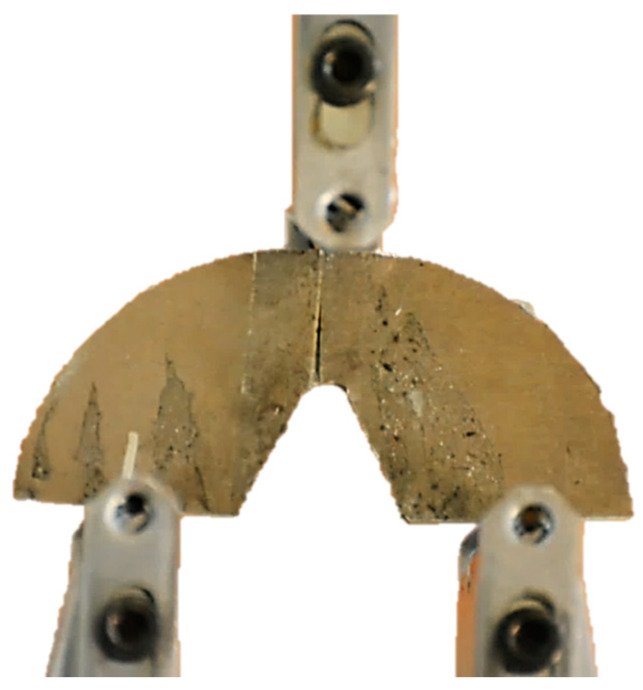
SCB notched sample under pure mode I fracture test.

**Figure 4:**
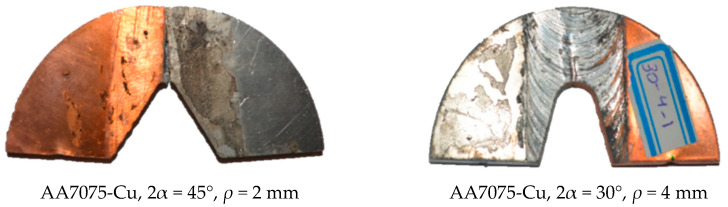

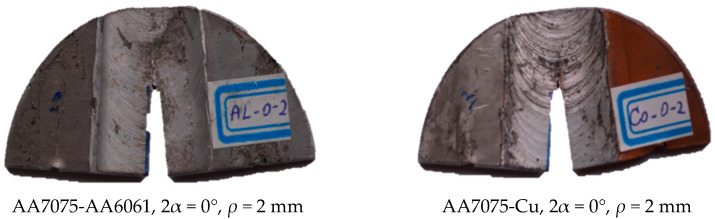
SCB notched samples after the pure mode I fracture test.

In order to determine the critical loads of the notched samples under mode I loading, they were quasi-statically loaded with a displacement rate of 1 mm/min in order to extract the force-displacement diagrams from the test machine. The testing machine utilized is an STM universal testing machine manufactured by SANTAM Corporation (Tehran, Iran), which is also equipped with an STM controller program for data acquisition. The loading with the rate of 1 mm/min is continued until a crack is initiated from the notch round tip and propagated to some extent, as it was observed that the load reached maximum values at the onset of the crack initiation. The main reason for not continuing the test until the final physical rupture is the instability of the process during the final crack propagation. This happens due to the significant out-of-plane plastic deformations, leading to the specimens falling from their place in the test machine. Figure 3 shows an SCB sample during the corresponding test, whereas Figure 4 depicts some of the specimens after being tested.

It is worth mentioning that, in order to accurately apply the load to the test specimens, and before their installation in the loading machine, the corresponding contact points of the loading pin and the supporting pins on the loading machine have been carefully determined and marked on each test specimen. Also, during the installation of the specimens in the machine, until they were fixed between the pins, they were kept and restrained by hand in the exact place.

### 2.2. Theoretical Models

Solutions for the mode I notch stress intensity factor (NSIF) in V-shaped notches have already been proposed for various notch opening angles and different notch tip radii [28,29]:

(1) $$K_{I}^{V,\rho }=\sqrt{2\pi }\frac{\sigma_{\theta \theta }\left(r_{0},0\right)r_{0}{}^{1-\lambda_{1}}}{1+\omega_{1}}$$

where *σ_θθ_* is the tangential stress and *r*_0_, *ω*_1_ and *λ*_1_ are geometrical parameters reported in [28,29].

According to the maximum tangential stress (MTS) criterion, the fracture of a notched component made of brittle or quasi-brittle materials occurs when the tangential stress at a specific critical distance, *r_c_*, from the notch tip (or a critical distance from the origin of the polar coordinates system, *r_c,V_*) equates the material critical stress, *σ_θθc_* [29]. The critical distance and the critical stress, as two material properties, are considered to be independent of the geometry and the loading conditions. *σ_θθc_* is normally considered equal to the material tensile strength, given that the final failure of brittle and quasi-brittle materials under tensile loading occurs when the inter-molecular bonds fail.

For estimating the mode I notch fracture toughness (NFT) according to the MTS criterion, the following closed form expression has already been suggested [29]:

(2) $$K_{Ic}^{V,\rho }=\frac{\sqrt{2\pi }\sigma_{\theta \theta }{}_{c}{\left(r_{0}+r_{c}\right)}^{1-\lambda_{1}}}{1+{\left(1+\frac{r_{c}}{r_{0}}\right)}^{\mu_{1}-\lambda_{1}}n_{\theta \theta }\left(0\right)}$$

where, again, *σ_θθc_* can be assumed to be equal to the ultimate tensile strength for most brittle and quasi-brittle materials. The values of *n_θθ_*(0), *λ*_1_, and *μ*_1_, which depend on the notch opening angle, have also been reported in [29]. *r_c,V_* is related with *r_c_* (the critical distance measured from the notch tip) via Equation (3):

(3) $$r_{c,V}=r_{0}+r_{c}=\frac{\pi -2\alpha }{2\left(\pi -\alpha \right)}\;\rho +r_{c}$$

where *r*_0_ is the distance between the origin of the coordinate system and the notch tip. Equation (4) specifies how *r_c_* is related to tensile and fracture properties [29]:

(4) $$r_{c}=\frac{1}{2\pi }{\left(\frac{K_{Ic}}{\sigma_{u}}\right)}^{2}$$

where *K_Ic_* and *σ_u_* are the material plain-strain fracture toughness and ultimate tensile strength, respectively.

Alternatively, according to the mean stress (MS) criterion, brittle fracture of a notched part takes place when the mean tensile tangential stress over a specific critical distance, *d_c_*, from the notch tip (or the critical distance from the origin of the polar coordinates system, *d_c,V_*) becomes equal to its critical value, *σ_θθc_*. The critical distance in the MS criterion can also be considered a material property.

For the estimation of the mode I NFT according to the MS criterion, the following closed form expression has been proposed [29]:

(5) $$K_{Ic}^{V,\rho }=\frac{\sqrt{2\pi }\sigma_{\theta \theta c}d_{c}}{\frac{1}{\lambda_{1}}\left[d_{c,V}^{\lambda_{1}}-r_{0}^{\lambda_{1}}\right]+\frac{n_{\theta \theta }\left(0\right)}{\mu_{1}r_{0}{}^{\mu_{1}-\lambda_{1}}}\left[d_{c,V}^{\mu_{1}}-r_{0}^{\lambda_{1}}\right]}$$

The relation determining the critical distance from the origin of the V-notch polar reference system is as follows:

(6) $$d_{c,V}=r_{0}+d_{c}=\frac{\pi -2\alpha }{2\left(\pi -\alpha \right)}\;\rho +d_{c}$$

where *d_c_* is the critical distance measured from the notch tip, which can be determined by the following Equation (7) [29]:

(7) $$d_{c}=\frac{2}{\pi }{\left(\frac{K_{Ic}}{\sigma_{u}}\right)}^{2}=4r_{c}$$

It is noteworthy that for sufficiently thin components, the plane-strain fracture toughness, *K_Ic_*, may be substituted by the corresponding fracture resistance, *K_c_* (i.e., that obtained using fracture specimens with the same thin thickness as the component being analyzed), in both Equations (4) and (7).

Now, concerning the Equivalent Material Concept (EMC), it states that a ductile material with a valid fracture toughness value (*K_Ic_* or *K_c_*) may be assumed to be equivalent to a virtual brittle material, provided their moduli of elasticity (*E*), fracture toughness values, and strain energy densities (SED) at the ultimate point of the tensile stress-strain curve are equal [30]. The SED of a material is the strain energy absorbed per unit volume of material. By combining the EMC with a brittle fracture criterion, linear elastic fracture analyses can be conducted to evaluate the ductile fracture of notched/cracked materials instead of using time-consuming and complex elastoplastic analyses [30].

By using the power-law stress-strain relation for the plastic behavior of an elastoplastic metallic material, and applying the EMC, Equation (8) has been obtained for the tensile strength of the equivalent brittle material.

(8) $$\sigma_{f}^{*}=\sqrt{\sigma_{Y}{}^{2}+\frac{2Ek}{n+1}\left(\varepsilon_{u,true}{}^{n+1}-0.002^{n+1}\right)}$$

where *n*, *k*, *E*, *ε_u,true_* and *σ_Y_* are the strain-hardening exponent, strain-hardening coefficient, elastic modulus, true plastic strain at the ultimate point, and yield strength, respectively. It is alternatively possible to derive the tensile strength of the equivalent material without the previous expression. To do so, the area under the stress-strain curve of the elastoplastic material until the ultimate point should be computed numerically and then be set equal to the area under the linear stress-strain curve of the equivalent brittle material [23,31].

As seen in Equations (2) and (5), in order to apply the MTS and the MS criteria for the prediction of the corresponding NFT, the critical distance from the notch tip, *r_c_* or *d_c_*, should be specified by Equations (4) and (7), respectively. To compute *r_c_* or *d_c_* for the equivalent material, *σ_u_* in Equations (4) and (7) should be substituted by σ_f_^*^. Consequently, for elastoplastic materials, these should be replaced by, respectively, Equations (9) and (10):

(9) $$r_{c}=\frac{1}{2\pi }{\left(\frac{K_{Ic}}{\sigma_{f}^{*}}\right)}^{2}$$

(10) $$d_{c}=\frac{2}{\pi }{\left(\frac{K_{Ic}}{\sigma_{f}^{*}}\right)}^{2}$$

## 3. Results and Discussion

**Figure 5:**
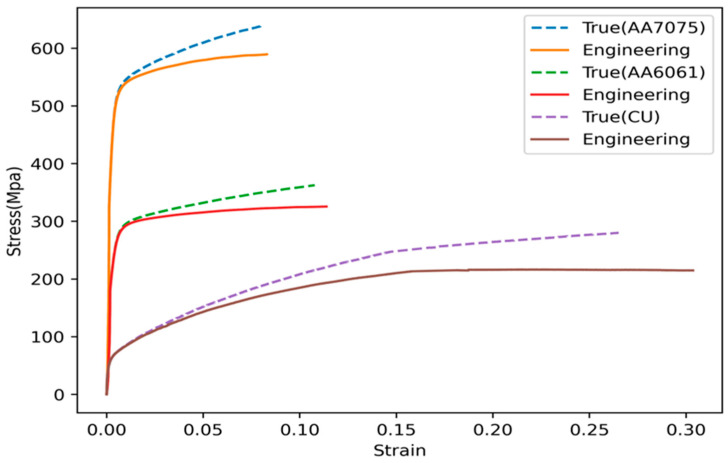
True and engineering stress-strain curves of AA7075-T6, AA6061-T6 and Cu materials.

**Figure 6:**
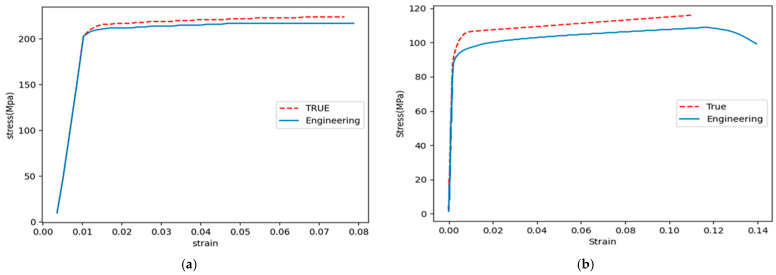
True and engineering stress-strain curves obtained for AA7075-AA6061 (**a**) and AA7075-Cu (**b**) weld materials.

The stress-strain diagrams of the three materials involved in this research are shown in Figure 5, whereas Figure 6 shows the tensile curves of the weld material obtained in the two FSWed joints being analyzed. Tests were performed following ASTM E8 standard (Standard Test Method for Tension Testing of Metallic Materials). The curves were plotted by using the data exported from the computer program of the testing machine and by means of Excel software (Microsoft Excel 2010). The resulting mechanical properties are listed in Table 1.

Furthermore, according to Figure 6, the modulus of elasticity for AA7075-AA6061 weld material is equal to 70.5 GPa, while this value for AA7075-Cu weld material is equal to 92 MPa.

**Figure 7:**
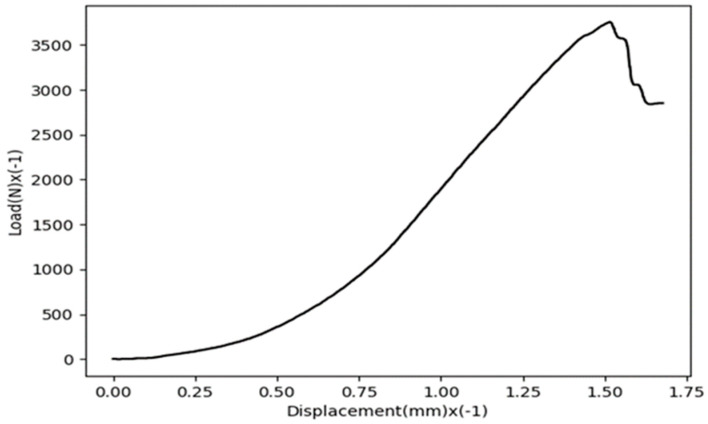
Load-displacement curve of a notched AA7075-AA6061 specimen with a notch angle of 30° and *ρ* = 1 mm.

The LBC values of the tested SCB specimens are listed in Table 2 and Table 3 for AA7075-AA6061 and AA7075-Cu joints, respectively. In these tables, the test specimens are coded. For example, sample code V45-1 refers to a V-notched specimen with a notch opening angle of 45° and a notch tip radius of 1 mm. The tables list the LBC of the individual tests, as well as the corresponding average value and average deviation for each combination of material and defect geometry. As an example, Figure 7 shows the load-displacement curve of a notched AA7075-AA6061 specimen with a notch angle of 30° and a notch tip radius of 4 mm. The curve is plotted by using the data exported from the computer program of the testing machine and by means of Python software (Python v3.9.2).

As mentioned before, using the EMC, in order to obtain the tensile strength of the equivalent material, the area under the true stress-strain curve of both the AA7075-AA6061 weld material (until the ultimate point) and the AA7075-Cu weld material is needed. After calculating such areas, and by equating them with the term σ_f_^*^^2^/2*E*, the equivalent material tensile strengths (σ_f_^*^) for AA7075-AA6061 and AA7075-Cu weld materials are 1450 MPa and 1150 MPa, respectively.

To obtain the NFT of the FSWed notched samples, first, the average critical loads of each combination of material and geometry were applied to their equivalent finite element models created in ABAQUS software (version 2019) together with the corresponding boundary conditions associated with the three-point bending test. Then, the linear elastic stress distributions in the specimens were obtained and the resulting mode I NSIFs were derived from Equation (1). These NSIFs are actually the NFTs (experimental) values, given that they were calculated for the critical loads.

**Figure 8:**
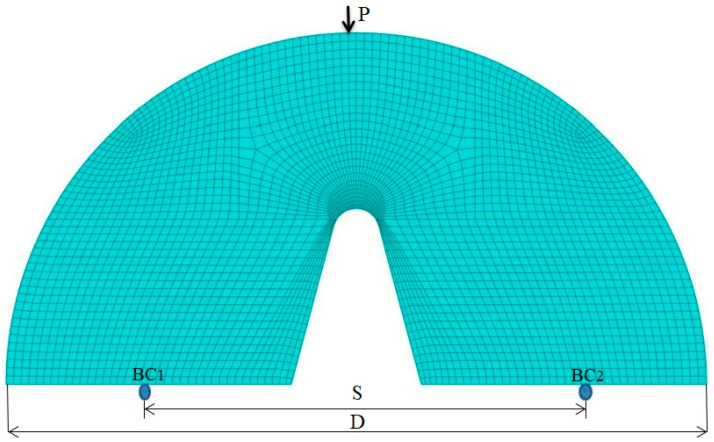
FE model of an SCB test specimen containing a blunt V-notch (2α = 30°).

Then, in order to use the MTS and MS criteria in combination with EMC to predict the NFTs of the notched AA6061-AA7075 samples with 2α = 0 and 45° and the notched AA7075-Cu samples with 2α = 30° and 60° (all samples with the notch tip radii of 1, 2 and 4 mm), first, the distribution of linear-elastic stresses in the SCB test specimens was determined using FE analyses (ABAQUS software). The loading and boundary conditions defined in the FE model of the SCB test specimen are shown in Figure 8.

In the FE model, reduced-integration quadratic elements were employed to mesh the model of the test samples. The element size was chosen based on the convergence of the results of FE analysis. As seen in Figure 8, very fine elements were utilized around the notch tip due to the high-stress concentration developed in that part of the samples.

In order to combine EMC with the brittle fracture criteria (MTS and MS), the critical distances *r_c_* and *d_c_* need to be defined. Then, the values of the equivalent material tensile strength (σ_f_*) and the fracture toughness for the AA7075-AA6061 and AA7075-Cu weld materials are required, according to Equations (9) and (10). σ_f_^*^ values were defined above (1450 MPa and 1150 MPa, respectively), while the values of *K_Ic_* have been reported in [24] and [31], with values of 42 MPa.m^0.5^ and 32 MPa.m^0.5^ for AA7075-AA6061 and AA7075-Cu, respectively. The resulting critical distances *r_c_* and *d_c_* are 0.13 mm and 0.52 mm for AA7075-AA6061 material, and 0.12 mm and 0.48 mm for the AA7075-Cu material. Now, Equations (2) and (5), with the critical distances defined by Equations (9) and (10), provide the NFTs estimations provided by the theoretical fracture criteria of EMC-MTS and EMC-MS, respectively. To make the theoretical NFT values comparable with the experimental ones, the critical loads of the notched samples given in Table 2 and Table 3 should be converted into the equivalent values of NFT. To do so, the critical loads of the samples were applied to the FE models, determining the value of the tangential stresses at the notch tip and using them in Equation (1). Table 4 presents the theoretical NFT values versus the experimental ones along with the discrepancies between them.

As observed in Table 4, the mean discrepancy between the experimental results and the predictions derived from the EMC-MTS criterion is close to 8%, whereas the mean discrepancy of the predictions derived from the EMC-MS criterion is 11.1%. Thus, the EMC-MTS criterion is slightly more accurate. This, together with the fact that the EMC-MTS criterion is simpler, suggests that this criterion could be more suitable to analyze the fracture behavior of notched FSWed AA7075-AA6061 and AA7075-Cu samples under mode I loading. This being said, in general, it can also be concluded that both the EMC-MTS and the EMC-MS criteria provide acceptable predictions.

In addition, the results shown in Table 4 reveal that all the theoretical predictions derived from the EMC-MS criterion, and most of the theoretical predictions derived from the EMC-MTS criterion, are larger than the corresponding experimental values. Therefore, it seems that both theoretical criteria overestimate the fracture resistance of the notched FSWed AA7075-AA6061 and AA7075-Cu samples under mode I loading. This overestimation requires further investigation in future works. At this point, it could be mentioned that its origin may be related to the simplifications associated with the use of the EMC and to the assumption of welds without significant defects (i.e., defects affecting the fracture behavior).

In order to determine the elastoplastic failure regime in the notched specimens examined, i.e., small-scale yielding (SSY), medium-scale yielding (MSY), large-scale yielding (LSY), or gross yielding (GY), the true stress and strain values in the plastic region of the stress-strain curves were first extracted. Then, by applying the critical load of each specimen to the associated FE model and using the true stress-strain curves, the size of the plastic zone around the notch and the corresponding failure regime of the notched sample were determined.

**Figure 9:**
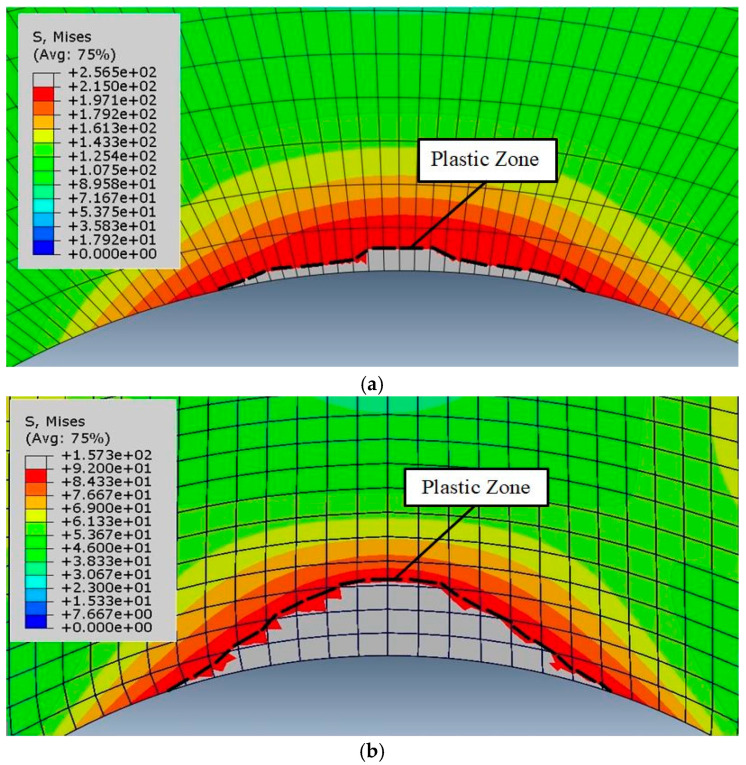
Plastic region around the notch tip for: AA7075-AA6061 weld material with 2α = 45° and ρ = 2 mm (**a**); AA7075-Cu weld material with 2α = 60° and ρ = 2 mm (**b**).

To ascertain the ligament size in the notched samples tested under three-point bending, first the tensile stresses and strains were determined and then, the effective ligament size was specified using linear elastic analysis. Eventually, by determining the plastic area in the notch vicinity at crack initiation and dividing its size by the effective ligament size, the borders of the plastic region and the elastoplastic failure regime were determined. Figure 9 shows two examples of the plastic region around the notch tip for the critical load and under pure mode I loading. Additionally, Table 5 specifies the elastoplastic failure regime for the tested samples.

It is worth mentioning that typically in literature, (PZS/ELS) × 100 values below 10 are regarded as SSY and 10–30 as MSY.

## 4. Conclusions

▪ The fracture of specimens made of the AA7075-AA6061 and AA7075-Cu FSW materials containing V-shaped notches and subjected to pure opening-mode loading was investigated.
▪ It was observed that for the specimens made of AA7075-AA6061 weld material, the elastoplastic fracture regime was small scale yielding (SSY), whereas for those made of AA7075-Cu, the fracture regime was moderate scale yielding (MSY).
▪ In order to utilize brittle fracture criteria for estimating the notch fracture toughness (NFT) values of the tested V-notched specimens, the EMC was employed.
▪ It was observed that the combination of EMC with the two brittle fracture criteria (maximum tangential stress—MTS criterion and mean stress-MS criterion) can result in successful predictions of the experimental results of the notched FSWed AA7075-AA6061 and AA7075-Cu materials subjected to mode I loading.
▪ Based on the results obtained, both the EMC-MTS and EMC-MS criteria provide acceptable predictions, but due to its simplicity, the EMC-MTS criterion is preferred here.

## Figures and Tables

**Table 1:** Mechanical properties of AA7075-T6, AA6061-T6, and Cu.

Material	*σ_Y_* (MPa)	*σ_u_* (MPa)	Strain at Failure (%)
AA7075	521	583	8
AA6061	276	292	11
Cu	58	230	27
AA7075- AA6061	215	220	7.8
AA7075-Cu	92	108	12

**Table 2:** LBC (maximum load) values obtained in the fracture tests of AA7075-AA6061 weld material.

Specimen	Test 1 (N)	Test 2 (N)	Test 3 (N)	Average (N) ± Avg. Deviation
V0-1	8845	6593	6972	7470 ± 917
V0-2	8195	9016	8321	8510 ± 337
V0-4	10,499	9400	10,001	9965 ± 378
V45-1	5839	6376	4601	5605 ± 670
V45-2	7077	6884	6795	6918 ± 106
V45-4	9067	9127	8817	9003 ± 124

**Table 3:** LBC (maximum load) values obtained in the fracture tests of AA7075-Cu weld material.

Specimen	Test 1 (N)	Test 2 (N)	Test 3 (N)	Average (N) ± Avg. Deviation
V30-1	3841	4130	4707	4226 ± 321
V30-2	5518	6070	5302	5630 ± 293
V30-4	7599	6639	6762	7000 ± 399
V60-1	3857	4589	4514	4320 ± 309
V60-2	6447	5607	5616	5890 ± 371
V60-4	7388	6335	7307	7010 ± 450

**Table 4:** Comparison between experimental and theoretical values of the NFT along with the discrepancies.

Test case	$K_{Ic}^{V,\rho }$ (Exp.)	$K_{Ic}^{V,\rho }$ (EMC-MS)	$K_{Ic}^{V,\rho }$ (EMC-MTS)	Discrepancy (%) (EMC-MS)	Discrepancy (%) (EMC-MTS)
AA7075-AA6061 0°—ρ1	52.7	58.1	50.9	10.2	−3.4
AA7075-AA6061 0°—ρ2	64.1	70.8	64.8	10.4	1.1
AA7075-AA6061 0°—ρ4	82.5	91.2	86.5	10.5	4.8
AA7075-AA6061 45°—ρ1	100.5	109.7	106	9.1	5.5
AA 7075-AA6061 45°—ρ2	133	149.4	146.7	12.3	10.3
AA7075-AA6061 45°—ρ4	186	206.7	204.7	11.1	10.1
AA7075-Cu 30°—ρ1	81.2	92.2	89.6	13.5	10.3
AA7075-Cu 30°—ρ2	114.3	126.6	124.7	10.8	9.1
AA7075-Cu 30°—ρ4	156.2	176.1	174.8	12.7	11.9
AA7075-Cu 60°—ρ1	71.9	81.8	78.8	13.8	9.6
AA7075-Cu 60°—ρ2	103.5	110.5	108.3	6.8	4.6
AA7075-Cu 60°—ρ4	134.3	151.9	150.4	13.1	11.9
Mean discrepancy (%)				**11.1**	**7.7**

**Table 5:** Elastoplastic failure regimes for the notched samples made of the FSWed materials. ELS: effective ligament size; PZS: Plastic zone size.

Case	ELS (mm)	PZS (mm)	(PZS/ELS) × 100 (%)	Failure Regime
AA7075-AA6061 $2\alpha =0^{{}^{\circ}}$, ρ = 1 mm	15	0.7	4.6	**SSY**
AA7075-AA6061 $2\alpha =45^{{}^{\circ}}$, ρ = 2 mm	15	0.5	3.3	**SSY**
AA7075-Cu $2\alpha =30^{{}^{\circ}}$, ρ = 2 mm	15	3.0	20	**MSY**
AA7075-Cu $2\alpha =60^{{}^{\circ}}$, ρ = 4 mm	15	2.8	18	**MSY**

## Data Availability

The data presented in this study are available on request from the corresponding authors.

## Nomenclature

*AA*	Aluminum alloy
*a*	Crack length
*d_c_*	Critical distance of the mean stress criterion measured from the notch tip
*d_c_^*^*	Critical distance of the mean stress criterion
*E*	Elastic modulus
*EMC*	Equivalent material concept
*FSW*	Friction-stir welding
*LBC*	Load bearing capacity
*LSY*	Large scale yielding
*k*	Strain-hardening coefficient
*K_I_*	Mode I notch stress intensity factor (NSIF)
*K_c_*	Fracture toughness
*K_Ic_*	Plane-strain fracture toughness
*MS*	Mean stress
*MSY*	Moderate scale yielding
*MTS*	Maximum tangential stress
*n*	Strain-hardening exponent
*NFT*	Notch fracture toughness
*NZ*	Nugget zone
*PS*	Point stress
*r_c_*	Critical distance of the MTS criterion measured from the notch tip
*r*_0_	Distance between the coordinate origin and the notch tip
*SED*	Strain energy density
*SCB*	Semi-circular bend specimen
*SSY*	Small scale yielding
*2α*	Notch rotation angle
*ε_u,true_*	True plastic strain at maximum load
*ρ*	Notch radius
*σ*	True stress
*σ_c_*	Critical stress
*σ_f_**	Tensile strength of the equivalent material
*σ_θθ_*	Tangential stress
*σ_θθc_*	Material critical stress
*σ_u_*	Ultimate tensile strength
*σ_Y_*	Yield strength

## References

[1] Sutton M.A., Reynolds A.P., Yang B., Taylor R. (2003). Mixed mode I/II fracture of 2024-T3 friction stir welds. Eng. Fract. Mech..

[2] Moreira P.M.G.P., Santos T., Tavares S.M.O., Richter-Trummer V., Vilaça P., de Castro P.M.S.T. (2009). Mechanical and metallurgical characterization of friction stir welding joints of AA6061-T6 with AA6082-T6. Mater. Des..

[3] Zadpoor A.A., Sinke J., Benedictus R. (2010). Global and local mechanical properties and microstructure of friction stir welds with dissimilar materials and/or thicknesses. Metall. Mater. Trans. A Phys. Metall. Mater. Sci..

[4] Reynolds A.P. (2002). R-curve behaviour of friction stir welds in aluminium-lithium alloy 2195. Fatigue Fract. Eng. Mater. Struct..

[5] Mokhtar S.N.F., Wahab A.A., Karuppanan S. (2012). Fracture toughness and fatigue crack growth study of friction stir welded plates. J. Appl. Sci..

[6] Sutton M.A., Reynolds A.P., Yang B., Taylor R. (2003). Mode I fracture and microstructure for 2024-T3 friction stir welds. Mater. Sci. Eng. A.

[7] Aliha M.R.M., Fotouhi Y., Berto F. (2018). Experimental notched fracture resistance study for the interface of Al–Cu bimetal joints welded by friction stir welding. Proc. Inst. Mech. Eng. Part B J. Eng. Manuf..

[8] Yan J., Sutton M.A., Reynolds A.P. (2006). Notch tensile response of mini-regions in AA2024 and AA2524 friction stir welds. Mater. Sci. Eng. A.

[9] Syafiq W.M., Rojan M.A., Abdul Majid M.S., Jaafar N.A. (2016). Fracture toughness of friction stir welded aluminium alloy. ARPN J. Eng. Appl. Sci..

[10] Fratini L., Pasta S., Reynolds A.P. (2009). Fatigue crack growth in 2024-T351 friction stir welded joints: Longitudinal residual stress and microstructural effects. Int. J. Fatigue.

[11] Pouget G., Reynolds A.P. (2008). Residual stress and microstructure effects on fatigue crack growth in AA2050 friction stir welds. Int. J. Fatigue.

[12] Bahemmat P., Besharati M.K., Haghpanahi M., Rahbari A., Salekrostam R. (2010). Mechanical, micro-, and macrostructural analysis of AA7075–T6 fabricated by friction stir butt welding with different rotational speeds and tool pin profiles. Proc. Inst. Mech. Eng. Part B J. Eng. Manuf..

[13] Aliha M.R.M., Shahheidari M., Bisadi M., Akbari M., Hossain S. (2016). Mechanical and metallurgical properties of dissimilar AA6061-T6 and AA7277-T6 joint made by FSW technique. Int. J. Adv. Manuf. Technol..

[14] Hatamleh O., Forth S., Reynolds A.P. (2009). Fatigue Crack Growth of Peened Friction Stir-Welded 7075 Aluminum Alloy under Different Load Ratios. J. Mater. Eng. Perform..

[15] Moreira P.M.G.P., de Jesus A.M.P., Ribeiro A.S., de Castro P.M.S.T. (2008). Fatigue crack growth in friction stir welds of 6082-T6 and 6061-T6 aluminium alloys: A comparison. Theor. Appl. Fract. Mech..

[16] Reynolds A.P., Tang W., Khandkar Z., Khan J.A., Lindner K. (2005). Relationships between weld parameters, hardness distribution and temperature history in alloy 7050 friction stir welds. Sci. Technol. Weld. Join..

[17] Alavi Nia A., Shirazi A. (2018). An investigation into the effect of welding parameters on fatigue crack growth rate and fracture toughness in friction stir welded copper sheets. Proc. Inst. Mech. Eng. Part L J. Mater. Des. Appl..

[18] Moghadam D., Farhangdoost K. (2016). Influence of welding parameters on fracture toughness and fatigue crack growth rate in friction stir welded nugget of 2024-T351 aluminum alloy joints. Trans. Nonferr. Met. Soc. China.

[19] Huang J., Meng Q., Zhan Z., Hu W., Shen F. (2019). Damage mechanics-based approach to studying effects of overload on fatigue life of notched specimens. Int. J. Damage Mech..

[20] Zhan Z., Ao N., Hu Y., Liu C. (2022). Defect-induced fatigue scattering and assessment of additively manufactured 300M-AerMet100 steel: An investigation based on experiments and machine learning. Eng. Frac. Mech..

[21] Torabi A.R. (2013). On the use of the equivalent material concept to predict tensile load-bearing capacity of ductile steel bolts containing V-shaped threads. Eng. Fract. Mech..

[22] Torabi A.R., Habibi R., Mohammad Hosseini B. (2015). On the ability of the equivalent material concept in predicting ductile failure of u-notches under moderate- and large-scale yielding conditions. Phys. Mesomech..

[23] Sutton M.A., Reynolds A.P., Yan J., Yang B., Yuan N. (2006). Microstructure and mixed mode I/II fracture of AA2524-T351 base material and friction stir welds. Eng. Fract. Mech..

[24] Torabi A.R., Kalantari M.H., Aliha M.R.M. (2018). Fracture analysis of dissimilar Al-Al friction stir welded joints under tensile/shear loading. Fatigue Fract. Eng. Mater. Struct..

[25] Torabi A.R., Mirzavand M., Saboori B. (2022). Investigation of notch effects on load-bearing capacity of AA7075-AA7075 friction-stir welded joints under mixed mode I/II loading. Theor. Appl. Fract. Mech..

[26] Torabi A.R., Saboori B., Mirzavand M. (2022). Elastoplastic fracture analysis of thin notched AA7075-AA2024 dissimilar friction-stir-welded plates under mixed mode I/II loading. Fatigue Fract. Eng. Mater. Struct..

[27] (2021). Standard Test Methods for Tension Testing of Metallic Materials.

[28] Lazzarin P., Filippi S. (2006). A generalized stress intensity factor to be applied to rounded V-shaped notches. Int. J. Solids Struct..

[29] Ayatollahi M.R., Torabi A.R. (2010). Brittle fracture in rounded-tip V-shaped notches. Mater. Des..

[30] Torabi A.R. (2012). Estimation of tensile load-bearing capacity of ductile metallic materials weakened by a V-notch: The equivalent material concept. Mater. Sci. Eng. A.

[31] Aliha M.R.M., Kalantari M.H., Ghoreishi S.M.N., Torabi A.R., Etesam S. (2019). Mixed mode I/II crack growth investigation for bi-metal FSW aluminum alloy AA7075-T6/pure copper joints. Theor. Appl. Fract. Mech..

